# *In vitro* Anti-viral Activity of Psoraleae Semen Water Extract against Influenza A Viruses

**DOI:** 10.3389/fphar.2016.00460

**Published:** 2016-11-30

**Authors:** Jang-gi Choi, Young-Hee Jin, Ji-Hye Kim, Tae Woo Oh, Nam-Hui Yim, Won-Kyung Cho, Jin Yeul Ma

**Affiliations:** Korean Medicine (KM) Application Center, Korea Institute of Oriental Medicine (KIOM)Daegu, South Korea

**Keywords:** Psoraleae semen water extract, influenza A virus, anti-viral activity, H1N1, H3N2

## Abstract

Influenza causes respiratory infections and poses health risks to humans and animals; its effects are complicated by increasing resistance to existing anti-influenza viral agents. Therefore, novel therapeutic approaches against influenza virus infection are required. Psoraleae semen has been widely used in traditional medicine in Korea, Taiwan, China, and Japan for treating and preventing various diseases. In this study, we examined the anti-viral activities and mechanism of action of the water extract of Psoraleae semen (WPS) using RAW 264.7 and MDCK cells. We found that pre- and post-treatment with 100 μg/mL WPS markedly inhibited influenza A virus replication as assessed using a green fluorescent protein reporter virus, reduced viral protein expression (NS-1, PA, HA, PB-1, M1, and M2), and inhibited NA and HA activities. Mechanism studies revealed that WPS induced type I interferon cytokine secretion and subsequent stimulation of an anti-viral state in RAW 264.7 cells. Further, WPS exerted inhibitory effects on neuraminidase in influenza virus strains H1N1 and H3N2. Meanwhile, WPS exhibited inhibitory effects on hemagglutination in H3N2 but not in H1N1. Based on these results, WPS serves as an immunomodulator and inhibitor of influenza hemagglutinin and neuraminidase. Our results suggest that WPS is a promising source of novel anti-influenza drug candidates.

## Introduction

Influenza viruses belong to the Orthomyxoviridae family. There are three types of influenza viruses: A, B, and C (Zambon, 1999). Influenza virus A can infect humans and different animals, including domestic and wild birds (Capua and Alexander, 2004). However, infection by type B and C viruses is mainly restricted to humans (Capua and Alexander, 2004). Influenza A virus (IAV) is the most serious influenza type as IAV infection can result in serious respiratory illness, respiratory complications, major economic loss, and death; additionally, the virus is particularly virulent among the elderly (Monto and Sullivan, 1993; Fleming et al., 2000; Fiore et al., 2009). Vaccines represent the most effective strategy for controlling influenza virus infection, but influenza vaccines have disadvantages, including inadequate protection, high cost, difficulty in predicting representative strains, and time requirements for design and production (Hayden, 2006; Fiore et al., 2009). Therefore, alternative strategies are required for efficiently controlling influenza outbreaks. There are two classes of anti-influenza drugs: neuraminidase (NA) inhibitors (oseltamivir, zanamivir, and peramivir) and adamantanes (amantadine and rimantadine; Meijer et al., 2014). However, high resistance levels have been reported for adamantanes against IAV, and these drugs are ineffective against influenza B virus (Centers for Disease and Prevention, 2006; Ison, 2011). NA inhibitors are widely used for treating and preventing influenza virus infection (Meijer et al., 2014). Numerous reports have illustrated that IAVs develop resistance to oseltamivir, zanamivir and peramivir (Centers for Disease and Prevention, 2006; Bouvier et al., 2008; Hurt et al., 2009). Further, NA inhibitors have several side effects (Kaji et al., 2005). Thus, there is a critical need to develop novel anti-influenza drugs to control and prevent future influenza pandemics and epidemics.

The innate immune system that includes type I interferons (IFNs) promotes innate anti-viral and anti-bacterial immunities (Anderson, 2000). Type I IFNs, namely α and β, are regulated by IFN regulatory factor (IRF)-7, IRF-3, NF-κB, and several intracellular signaling molecules, which are activated by germline-encoded pattern recognition receptors recognizing the molecular pattern specific to microorganisms (Marié et al., 1998; Sato et al., 2000). During viral infection, rapid IFN production is needed to prevent the spread of the virus in the host. Medicinal plants have been the most important sources of medicine worldwide for some time, and they will be continue to serve as sources of novel remedies for humans. Medicinal plants exhibit numerous beneficial therapeutic properties, and it is thought that the mechanisms involved in these effects are due to the modulation of innate immunity. In particular, a lot of extracts or substances from medicinal herbs or plants have anti-viral effects against infectious viruses (Cowan, 1999; Cho et al., 2015). Therefore, natural product or standardized plant extracts provide unlimited opportunities for new anti-viral drugs with high efficacies, low toxicities, and minor side effects.

Psoraleae semen (PS) has been widely used in traditional Korean, Chinese, and Asian herbal medicine to treat various diseases and conditions such as several skin diseases including leukoderma, cardiovascular diseases, nephritis, osteoporosis, cancer, and hair loss (Khushboo et al., 2010; Zhang et al., 2016). Additionally, PS extracts and active components possess many pharmacological and biological activities, including, estrogenic, anti-microbial, anti-depressant, anti-inflammatory, anti-oxidant, hypotensive, osteoblastic, hepatoprotective, and anti-tumor activities (Zhang et al., 2016). The main PS components include flavonoids, coumarins, and meroterpenes, and the major active compounds are bakuchiol, bavachin, angelicin, psoralen, and corylifolin (Zhang et al., 2016). Psoralen inactivates influenza viruses, herpes simplex viruses, and vesicular stomatitis virion RNA polymerase activity (Nakashima et al., 1979; Redfield et al., 1981), and angelicin derivatives inhibit influenza virus (Yeh et al., 2010). Recently, bakuchiol was shown to have anti-influenza viral activity by Nrf2 activation (Shoji et al., 2015). Consequently, a PS extract could be a candidate with anti-influenza virus activity. We hypothesize that water extract of PS (WPS) induces an anti-viral state in murine macrophage cells (RAW 264.7) via the modulation of the immune response, induction of anti-viral cytokines, and overall inhibition of virus replication.

Here we investigated WPS-induced signaling molecules and confirmed the immunomodulatory potential of these molecules to regulate the innate immune response of PS, which may be responsible for its anti-viral activity in murine macrophage cells. Further, we evaluated PS efficacy against IAVs *in vitro*. Finally, prophylactic WPS efficacies against divergent influenza A subtypes, including H1N1 and H3N2, were assessed.

## Materials and Methods

### WPS Preparation

The PS identity obtained from Yeongcheon Oriental Herbal Market (Yeongcheon, Korea) was first certificated by Professor Ki Hwan Bae (College of Pharmacy, Chungnam National University, Daejeon, Korea), and PS was stored in the KM-Application Center herbarium, Korea Institute of Oriental Medicine. To prepare WPS, dried PS (50 g) was soaked in distilled water (1 L) and then heat-extracted at 115°C for 3 h in a Cosmos-600 Extractor (Gyeonseo Co., Incheon, Korea). After filtration through standard testing sieves (150 μm, Retsch, Haan, Germany), WPS was freeze-dried and stored in desiccators at 4°C. The amount of WPS powder collected was 15.6 g, and the yield was 12.96%.

### Cells and Viruses

RAW 264.7 and MDCK cells were obtained from the American Type Culture Collection (Manassas, VA, USA). Cells were maintained in DMEM or RPMI 1640 (Lonza, Walkersville, MD, USA) supplemented with 10% (v/v) heat-inactivated fetal bovine serum (Cellgro, Manassas, VA, USA) and penicillin (100 U/mL)/streptomycin (100 μg/mL) at 37°C with 5% CO_2_. Influenza A/PR/8/34 (H1N1) and green fluorescent protein (GFP)-tagged H1N1 (H1N1-GFP) were donated by Dr. Jong-Soo Lee, College of Veterinary Medicine, Chungnam National University. Influenza A strains H3N2 (KBPV-VR-32) and H1N1 (KBPV-VR-33) were obtained from Korea Bank for Pathogenic Viruses. H1N1, H3N2, and H1N1-GFP were propagated in the allantoic fluid of 10-day-old chicken embryos.

### Reagents and Antibodies

Recombinant mouse IFN-β, ribavirin, zanamivir, and lipopolysaccharides from *Escherichia coli* were obtained from Sigma Chemical Co. (St. Louis, MO, USA). Anti-IRF3, anti-phospho-IRF3 anti-STAT1, anti-phospho-STAT1, anti-TBK1, and anti-phospho-TBK1 antibodies were obtained from Cell Signaling Technology (Boston, MA, USA), and anti-β-actin was purchased from Santa Cruz Biotechnology (Santa Cruz, CA, USA). Antibodies against NS-1, PA, HA, PB-1, M2, and M1 were purchased from GeneTex (San Antonio, TX, USA).

### Viral Replication Inhibition Assay

A GFP-based viral replication assay was performed with a GFP-tagged IAV as described previously (Talactac et al., 2015). RAW 264.7 cells were cultured in six-well plates (1 × 10^6^ cells/well) for 12 h. Cells were then exposed to a medium (RPMI, negative control), 1000 U recombinant mouse IFN-β (positive control), or 100 μg/mL WPS. After 12 h, cells were infected with H1N1-GFP [multiplicity of infection (MOI) = 1]. GFP expression was observed under a microscope after 24 h of virus infection. Cell viability was determined via the MTS or CCK-8 assay (pre-treatment method). Additionally, for post-treatment analysis, MDCK cells were seeded onto a 96-well plate and infected with H1N1 for 2 h, after which the virus was removed and cells were treated with WPS at 100 or 400 μg/mL for 24 h. The medium was the negative control, and ribavirin (10 μg/mL) was the positive control. Virus-induced cell death was measured by the MTS or CCK-8 assay.

### Viral Yield Reduction Assay

To determine the ability of WPS to inhibit virus-induced red blood cell (RBC) hemolysis, RAW 264.7 cells were seeded in six-well plates at 1 × 10^6^ cells per well and were incubated overnight to reach 70% confluence. Cells were washed with PBS and infected with H1N1-GFP (MOI = 1). The virus yield reduction assay was performed after 24 h of incubation with WPS. The medium was the negative control, and IFN-β was the positive control. Briefly, 50 μL PBS was added to each well of a U-bottomed 96-well plate. The infected supernatant, WPS, IFN-β, and medium were serially diluted twofold in the previously loaded PBS. Finally, 100 μL 1% chicken RBCs were added to each well. Assays were evaluated for 1 h of incubation at room temperature. RBCs in negative wells sedimented and exhibited agglutination, whereas positive wells had an opaque appearance or displayed hemolysis with no sedimentation. Titers are presented in HA units/50 μL (HAU/50 μL) in comparison with the control treatment (Eisfeld et al., 2014; Abdal Dayem et al., 2015).

### Real-time RT-PCR

Total RNA was extracted using RNeasy Mini Kit (QIAGEN, Valencia, CA, USA) according to the manufacturer’s instructions. RNA concentrations were measured using a NanoDrop ND-1000 spectrophotometer (NanoDrop Technologies, Wilmington, DE, USA), and total RNA (1 μg) was converted to cDNA using RevoScript^TM^ RT PreMix (iNtRON Biotechnology, Sungnam, Korea). Real-time RT-PCR was performed using the primers listed in **Table 1**. Reactions were conducted in triplicate with a total volume of 20 μL consisting of 0.3 μM of each primer, 10 μL AccuPower^®^ 2X Greenstar qPCR Master Mix (Bioneer, Daejeon, Korea), and 2 μL template DNA. Amplification and analyses were performed using QuantStudio 6 Flex Real-Time PCR System (Thermo Scientific). Relative expression was calculated using the ΔΔCt method. Individual transcripts in each sample were assayed three times and normalized to GADPH mRNA levels.

**Table 1:** Primers sequences for real-time RT-PCR.

Name	Orientation	Primer sequences 5′ to 3′ orientation
GAPDH	Forward	TGACCACAGTCCATGCCATC
	Reverse	GACGGACACATTGGGGGTAG
STAT1	Forward	TGGTGAAATTGCAAGAGCTG
	Reverse	CAGACTTCCGTTGGTGGATT
IRF-7	Forward	AAGCTGGAGCCATGGGTATG
	Reverse	GACCCAGGTCCATGAGGAAG
IFN-beta	Forward	TCCAAGAAAGGACGAACATTCG
	Reverse	TGCGGACATCTCCCACGTCAA
ISG-20	Forward	AGAGATCACGGACTACAGAA
	Reverse	TCTGTGGACGTGTCATAGAT


### Hemagglutination Assay

WPS was diluted (1–1000 μg/mL) with PBS in a round-bottomed 96-well plate. The medium was used as a negative control. H1N1, H1N1-GFP, or H3N2 (64 HAUs in PBS) was added to each well and incubated for 30 min at 37°C under 5% CO_2_. Each sample was mixed with 1% chicken RBCs (Innovative Research, Inc., Southfield, MI, USA) in PBS. After incubation for 1 h at room temperature, plates were photographed.

### NA Inhibition Assay

WPS was used at 1–2000 μg/mL. Zanamivir, a specific NA blocker, was used as a control at 0.001–10 μg/mL. The NA inhibition assay was performed according to the manufacturer’s instructions using the NA-Fluor Influenza Neuraminidase Assay kit (Life Technologies, Carlsbad, CA, USA). The reaction was monitored using a fluorescence spectrometer in the kinetic mode at an excitation wavelength of 365 nm and emission wavelength of 445 nm (Abdal Dayem et al., 2015).

### Western Blot Analysis

Total protein expression of whole cell extracts or cytoplasmic and nuclear extracts was determined using Bradford reagent (Bio-Rad, Hercules, CA, USA). Equal protein amounts were resolved on sodium dodecyl sulfate gels by polyacrylamide gel electrophoresis and transferred to PVDF membranes. After blocking non-specific sites with 5% skim milk, membranes were incubated with each primary (1:1000 dilution) and secondary (1:2000 dilution) antibody, and expression was detected using SuperSignal West Femto Chemiluminescent Substrate (Thermo Scientific). Relative band intensity was measured using ImageJ.

### Enzyme-Linked Immunosorbent Assay

Murine IL-6, TNF-α, and IL-1β levels in culture supernatants were determined using enzyme-linked immunosorbent assay antibody kits (eBioscience, San Diego, CA, USA) according to the manufacturer’s instructions.

### Immunocytochemistry

RAW 264.7 cells (0.5–1.0 × 10^5^) grown on a four-well tissue culture slide were cultured at 37°C for 24 h. For pre-treatment, WPS (100 μg/mL) was added to cells, which were incubated in a CO_2_ incubator at 37°C for 12 h. After WPS treatment, the medium was discarded, and cells were washed with PBS and infected with H1N1 (MOI = 1) for 2 h. After infection, the medium was discarded and cells were washed with PBS; the medium was then added to cells, which were incubated in a CO_2_ incubator at 37°C for 24 h. For post-treatment with WPS, cells were infected with H1N1 (MOI = 1) for 2 h. After infection, the medium was discarded, and cells were washed with PBS. Next, WPS was added to cells, which were incubated in a CO_2_ incubator at 37°C for 24 h. At 24 h post-infection, cells were washed with PBS three times and fixed with 4% paraformaldehyde for 30 min and 1% Triton X-100 at room temperature. After blocking, fixed cells were incubated with an M2-specific antibody overnight, washed with TBS three times, and incubated with Alexa Fluor 568-goat anti-rabbit IgG antibody (1:1000; Life Technologies, Eugene, OR, USA) for 1 h at 25°C. Next, cells were incubated with DAPI for 10 min and observed by fluorescence microscopy.

### High-Performance Liquid Chromatography (HPLC) Analysis

Water extract of PS standardization was performed by HPLC fingerprinting with chemical standards purchased from ChemFaces (psoralen, angelicin, bavachin, and bavachinin; Wuhan, Hubei, China). Standard solutions were prepared by dissolving each marker component in 100% methanol at 1 mg/mL. WPS powder was accurately weighed and dissolved in methanol at 10 mg/mL for analysis. HPLC-grade acetonitrile was purchased from Merck (Darmstadt, Germany), and acid was purchased from Sigma-Aldrich. Separation was performed in a Dionex HPLC system (Dionex Co., Sunnyvale, CA, USA) equipped with an ultimate 3000 series A binary pump, an auto-sampler, a column oven, and a diode array UV/VIS detector. Data analysis was performed using Dionex Chromeleon. All chromatographic separations were performed on Acclaim C18 (4.6 × 250 mm^2^, 5 μm, Dionex) at 30°C. HPLC analysis was performed in accordance with the methods reported by Zhao et al. (2005) with some modifications. Briefly, the mobile phase consisted of water containing (A) 0.1% acetic acid and (B) acetonitrile with gradient elution at a flow rate of 1 mL/min. Gradient elution was performed as follows: 40–50% (v/v) B at 0–15 min, 50–60% B at 15–35 min, 60–70% B at 35–45 min, 70–80% B at 45–55 min, and 80% B at 55–60 min. The injection volume was 10 μL, and detection wavelength was 245 nm.

### Statistics

All data are presented as mean ± SD of at least three independent experiments. Statistical significance of mean values in the two groups or treatment effects were evaluated via one-way ANOVA with Dunnett’s test. Analyses were performed using GraphPad PRISM software^®^ (GraphPad PRISM software Inc., Version 5.02, La Jolla, CA, USA). Values of ^∗^*P* < 0.05, ^∗∗^*P* < 0.005, and ^∗∗∗^*P* < 0.0005 indicated statistical significance.

## Results

### Effects of WPS on Cell Viability

**FIGURE 1:**
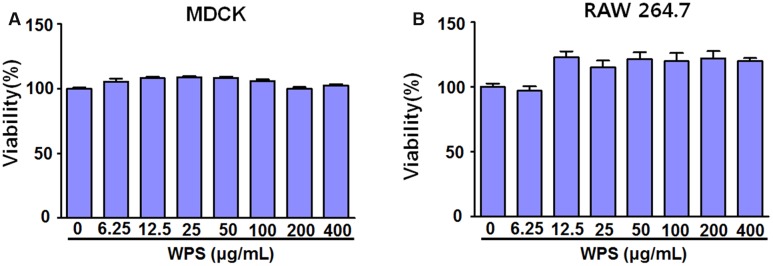
**Determination of the effective cytotoxic concentration of the water extract of Psoraleae semen (WPS) in** **(A)** MDCK and **(B)** RAW 264.7 cells. The indicated concentrations of WPS were added to MDCK and RAW 264.7 cells. Cell viabilities were determined by the CCK-8 or MTS assay after 24 h (*n* = 3 each). Data are representative of three independent experiments.

WPS cytotoxicities in RAW 264.7 and MDCK cells were evaluated using the CCK-8 assay after 24 h treatment to determine the optimal concentration that would provide anti-viral activity with minimum toxicity. The results illustrated that WPS concentrations of ≤400 μg/mL had no significant effects on cell viability, indicating that WPS was not toxic to RAW 264.7 and MDCK cells (**Figures 1A,B**). Therefore, subsequent experiments were performed using concentrations of ≤100 μg/mL.

### WPS Increased RAW 264.7 Cell Survival

**FIGURE 2:**
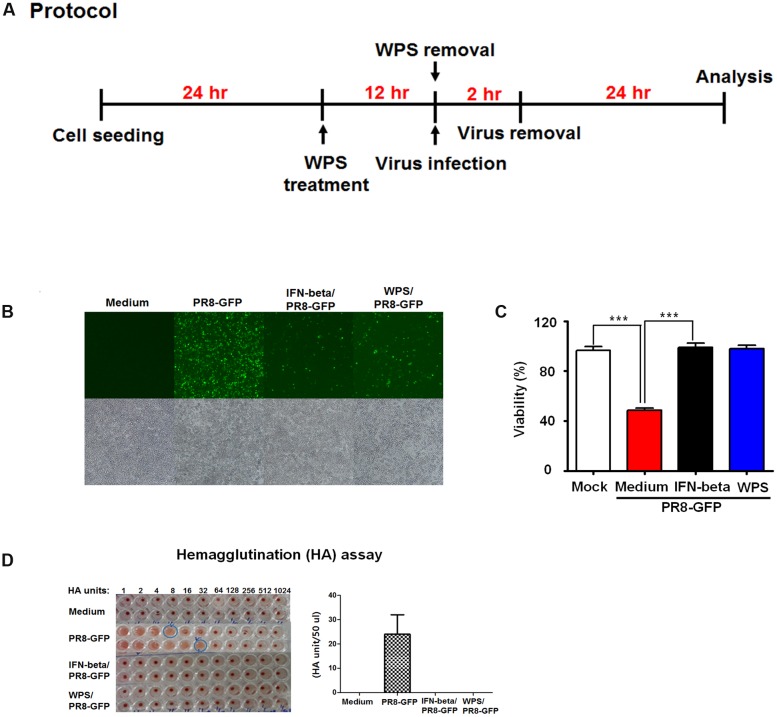
**Anti-viral activities of pre-treatment with WPS on the influenza A/PR/8/34 (H1N1)-GFP virus in RAW 264.7 cells.** **(A)** RAW 264.7 cells were treated with WPS prior to influenza A virus infection. **(B)** Cells incubated with the medium alone, 100 μg/mL WPS, or 1000 U/mL interferon-β (recombinant mouse) 12 h prior to infection with H1N1-GFP (multiplicity of infection = 1). Images of GFP expression were obtained 24 h after virus infection **(C)** Cell viability was determined 24 h after virus infection by the MTS or CCK 8 assay, and survival is presented as a percentage of the control value (cells without treatment). Data are presented as the mean ± SD (error bars) of three independent experiments. ^∗∗∗^*P* < 0.0005 indicates a significant difference between groups. **(D)** Viruses were titrated from the supernatant via the hemagglutination assay. Error bars indicate the range of values obtained from two independent experiments.

To evaluate anti-influenza virus activity of WPS, viral replication using GFP-expressing viruses was examined in cells pre-treated with WPS (**Figure 2A**). H1N1-GFP-infected RAW 264.7 cells with pre-treated WPS exhibited markedly reduced GFP expression compared to that in the untreated group (**Figure 2B**). **Figure 2C** shows that cell death significantly decreased in WPS-treated cells compared with that in the virus-only group within 24 h of H1N1-GFP infection. We found that viral supernatant titers of H1N1-GFP-, IFN-beta/H1N1-GFP-, and WPS/H1N1-GFP-infected cells were significantly reduced by WPS or IFN-β treatment (0 HAUs) compared to that in the virus-only group (64 HAUs; **Figure 2D**). These results illustrate that WPS pre-treatment reduced H1N1-GFP replication in RAW 264.7 cells.

### WPS Induced IFN-β and Pro-inflammatory Cytokine Secretion and Type I IFN Signaling Pathway Activation in RAW 264.7 Cells

**FIGURE 3:**
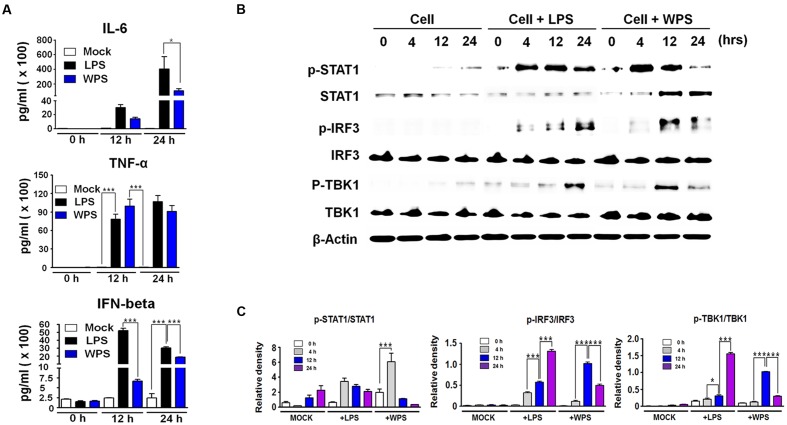
**Stimulation of an anti-viral state by WPS in RAW 264.7 cells.** Cells were treated with the medium alone (mock), 100 ng/mL lipopolysaccharides, or 100 μg/mL WPS and were then incubated at 37°C and 5% CO_2_. Cells treated with the medium served as the negative controls. The supernatant from each group was harvested at 0, 12, and 24 h and clarified by centrifugation at 3500 rpm for 20 min at 4°C. **(A)** The cytokines (IL-6, TNF-α, and IFN-β) secreted in treated RAW 264.7 cells were measured by an enzyme-linked immunosorbent assay using antibody-coated enzyme-linked immunosorbent assay plates. The test was performed in triplicate. The data show representative means ± SDs of each murine cytokine measured over time from three independent assays. Asterisks indicate a significant difference between groups (^∗∗∗^*P* < 0.0005, ^∗^*P* < 0.05). **(B,C)** Immunoblot analysis was performed using the lysates of RAW 264.7 cells treated with or without WPS to assess the expression of the phosphorylated and non-phosphorylated forms of STAT1, IRF3, TBK1, and β-actin over time. The experiment was repeated three times independently, and similar results were obtained. ^∗∗∗^*P* < 0.0005, ^∗^*P* < 0.05 compared with the mock value.

The anti-viral response of WPS may be related to the innate immune response through the expression of cytokines, such as IL-6 and IFN-β. To correlate these observations with the IFN-inducing signaling pathway, we examined the phosphorylation of interferon-related signaling molecules. For elucidating the possible mechanism by which WPS inhibited viral replication, we first measured secreted cytokine levels (IFN-β, IL-6, and TNF-α) in treated and untreated virus-infected RAW 264.7 cells. As shown in **Figure 3A**, WPS induced high levels of IL-6, TNF-α, and IFN-β secretion (**Figure 3A**) after 24 h compared to levels in medium- or lipopolysaccharide-treated positive controls. The results indicate that IL-6, TNF-α, and IFN-β can be induced by WPS, thus mediating the anti-viral state in RAW 264.7 cells. In addition, the anti-viral effect of WPS may be related to the innate immune response through cytokine expression, such as IFN-β and IL-6. To correlate the aforementioned observations with the IFN signaling pathway, we examined the effects of WPS on type I IFN-related protein phosphorylation. For this purpose, whole cell lysates of WPS-treated RAW 264.7 cells were subjected to immunoblotting to analyze the expression of the phosphorylated and non-phosphorylated forms of STAT1, IRF3, and TBK1. As shown in **Figure 3B**, WPS treatment in RAW 264.7 cells upregulated phosphorylation of STAT1, IRF-3, and TBK1, which are important molecules in the type I IFN signaling pathway (Trinchieri, 2010). IRF3 phosphorylation indicates IRF3 molecule translocation into the nucleus and transcription initiation of type I IFNs. Consequently, the produced type I IFNs bind to proteins in JAK-STAT pathway (Horvath, 2004; Trinchieri, 2010), leading to the phosphorylation of STAT1 and transcriptional activation of interferon-stimulating genes (ISGs). Our results indicate that WPS treatment induces IRF3, STAT1, and TBK1 phosphorylation at 12 h to levels compared to findings in untreated cells (**Figures 3B** and **2C**).

**FIGURE 4:**
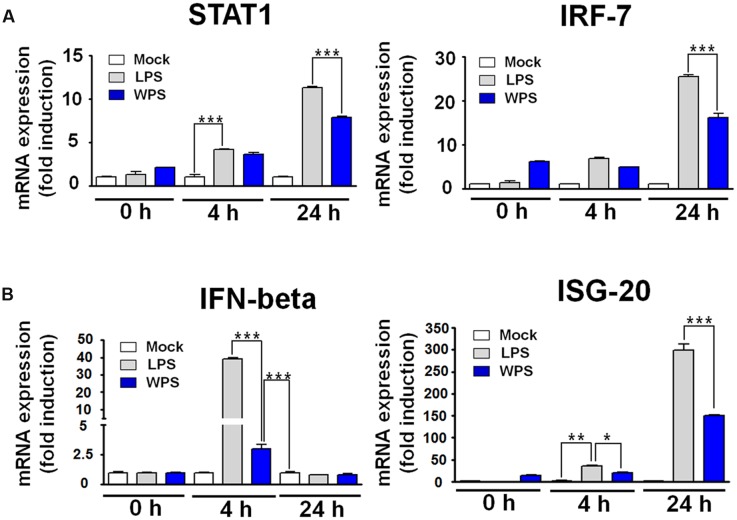
**Induction of interferon (IFN)-related gene and IFN-stimulating gene (ISG) transcripts by WPS in RAW 264.7 cells.** Cells were treated with the medium alone (mock), 100 ng/mL lipopolysaccharides, or 100 μg/mL WPS and were then incubated at 37°C with 5% CO_2_. The time-dependent changes in mRNA expression (**A,B**; STAT1, IFN-β, IRF-7, and ISG-20) were examined after treatment in RAW 264.7 cells. ^∗∗∗^*P* < 0.0005, ^∗∗^*P* < 0.005, and ^∗^*P* < 0.05 compared with the mock value.

We further confirmed the interaction between WPS and IFN-stimulated gene induction in RAW 264.7 cells. A time-dependent increase in the mRNA expression of STAT1, IRF-7, IFN-β, and ISG-20 was observed in WPS-treated RAW 264.7 cells compared with levels in untreated cells (**Figure 4A**). In addition, upregulation of IFN-β and ISG was noted at 24 h. The observed pattern was similar to that of lipopolysaccharide-treated positive control (**Figure 4A**), and transcription of IRF-7 and ISG-20 increased by 16- and 150-fold, respectively. The overall results suggest that WPS induces the anti-viral state by modulating the IFN signaling pathway and IRF-7 and ISG-20 expression in RAW264.7 cells, which may result in viral replication inhibition (Espert et al., 2005; Lazear et al., 2013; Sheikh et al., 2014).

### WPS Inhibited H1N1 Infection in MDCK Cells

**FIGURE 5:**
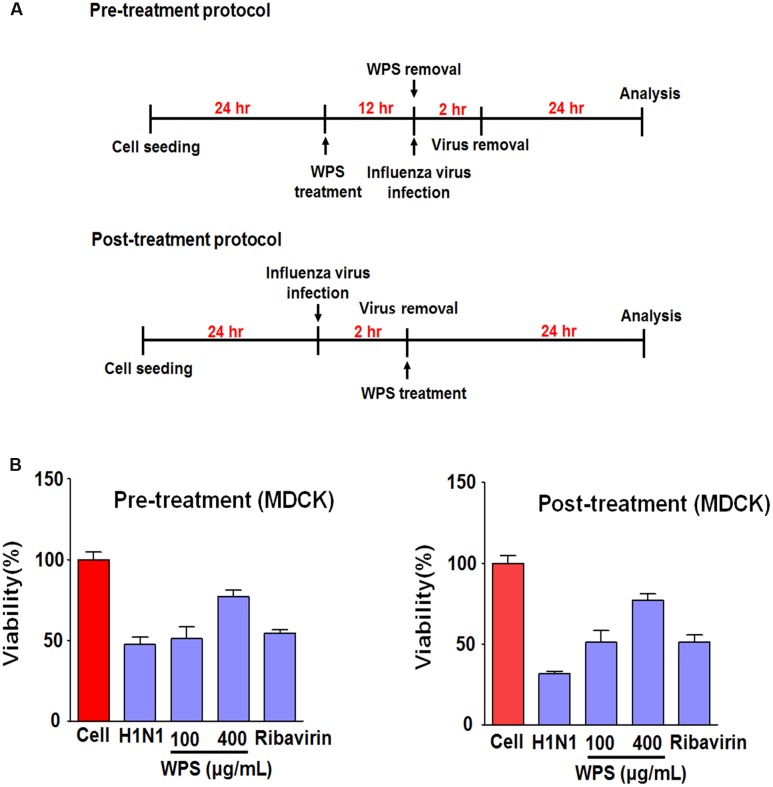
**WPS inhibited influenza A/PR/8/34 (H1N1) infection in MDCK cells.** **(A)** MDCK cells were treated with WPS before or after viral adsorption. **(B)** Cells were incubated with the medium alone (mock), 100 μg/mL WPS, or 10 μg/mL ribavirin before or after infection with H1N1-GFP (multiplicity of infection = 1). Cell viability was determined 24 h after viral infection by the MTS or CCK 8 assay, and survival is presented as a percentage of the control value (untreated cells). Data are presented as the mean ± SD (error bars) of three independent experiments.

To investigate whether WPS inhibited IAV infection in MDCK cells, we examined viral replication in MDCK cells treated with WPS before and after infection with H1N1 (**Figure 5A**). WPS-pre-treated and WPS-post-treated (100 and 400 μg/mL) cells displayed significantly decreased cell death compared to that in cells exposed only to H1N1 (**Figure 5B**), indicating that both pre- and post-treatment with WPS reduces viral replication in MDCK cells.

### WPS Reduced H1N1 Viral Protein Expression

**FIGURE 6:**
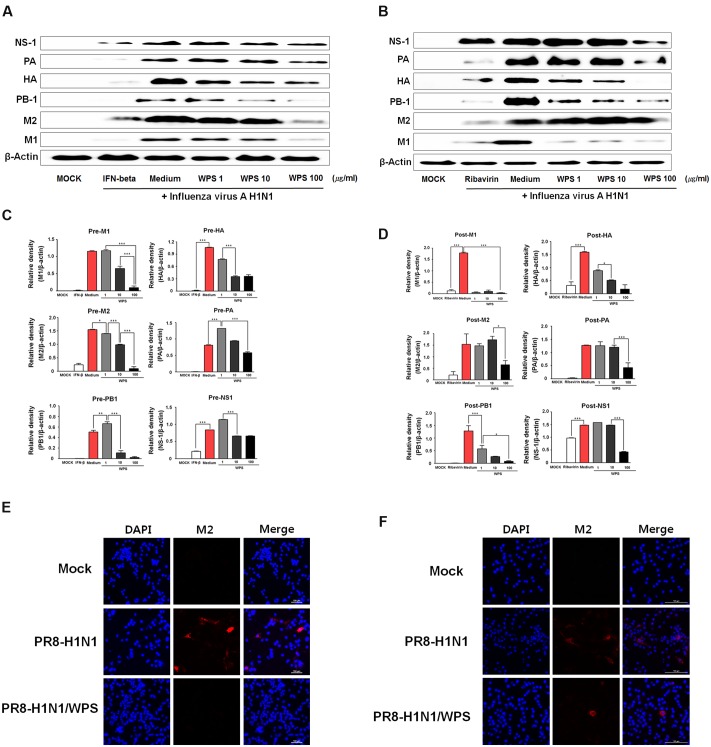
**WPS inhibited influenza A/PR/8/34 (H1N1) protein expression in RAW 264.7 cells.** RAW 264.7 cells were treated with WPS (100 μg/mL), interferon (IFN)-β (1000 U/mL, recombinant mouse, pre-treatment positive control), ribavirin (10 μg/mL, post-treatment positive control), or the medium only (negative control) before **(A,C)** or after **(B,D)** after viral adsorption. Influenza A virus protein levels (NS-1, PA, HA, PB-1, M1, M2) in cell lysates were analyzed by Western blotting, and β-actin expression was analyzed as an internal control. Data are presented as the mean ± SD (error bars) and are representative of three independent experiments. ^∗∗∗^*P* < 0.0005, ^∗∗^*P* < 0.005, ^∗^*P* < 0.05 compared with the mock value. RAW 264.7 cells were infected with H1N1 (multiplicity of infection = 1) before **(E)** or after WPS treatment **(F)**. Cells were observed by fluorescence microscopy using an M2-specific antibody. The cells were also stained with DAPI. The merged image illustrates the cytoplasmic localization of viral M2 (red) and selected nuclei (DAPI, blue).

To evaluate whether WPS inhibited H1N1 viral protein expression, we performed Western blotting in RAW 264.7 cells treated with WPS before and after H1N1 infection. Relative protein expression levels of viral proteins (PA, HA, PB-1, M2, M1) as analyzed by Western blotting were significantly lower in RAW 264.7 cells with pre- or post-treatment of WPS than in those treated with the medium alone (**Figures 6A,B**). WPS pre-treatment decreased M1, M2, and PB1 levels by 0.8-, 0.9-, and 0.9-fold, respectively. Post-treatment with WPS decreased M1, HA, and PB1 levels by 0.9-, 0.4-, and 0.9-fold, respectively (**Figures 6C,D**). Further, we demonstrated, by immunofluorescent analysis, that M2 protein levels significantly decreased in RAW 264.7 cells treated with WPS before and after H1N1 exposure compared with levels in untreated cells (**Figures 6E,F**). Therefore, these data revealed that pre- and post-treatment with WPS reduced H1N1 virus protein expression.

### Inhibitory Effects of WPS on NA Activity

**FIGURE 7:**
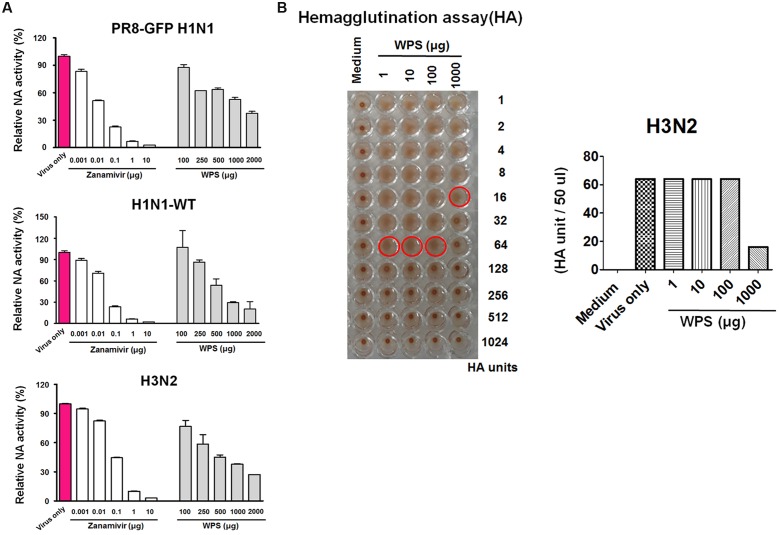
**Measurement of the anti-viral activity of WPS using neuraminidase inhibition and hemagglutination inhibition assays.** **(A)** Influenza A viruses (H1N1 and H3N2) were added at 32 hemagglutination units (HAUs) to the indicated concentrations of WPS, zanamivir (positive control), or PBS (negative control); were mixed with NA-Fluor^TM^ substrate; and were incubated at 37°C for 1 h away from light. Fluorescence was monitored (excitation, 365 nm; emission, 445 nm). Data are representative of two independent experiments, and results were reproducible. **(B)** Hemagglutination inhibition assay: WPS was serially diluted using PBS and added to equal volumes of the viruses (64 HAUs). To assess red blood cell (RBC) hemolysis inhibition, 50 μL 1% chicken RBCs were added to each well of a 96-well plate and incubated for 1 h at room temperature. Data are representative of two independent experiments, and results were reproducible.

NA is a key viral protein responsible for releasing newly produced virus particles and the recognition and cleavage of target cell receptor sialic acid moieties (N-acetylneuraminic acid) on infected cells (Chazal and Gerlier, 2003). Additionally, NA activity is required for preventing the self-aggregation of virus particles via the cleavage of sialic acids bound to the virus surface. We therefore tested the potential effect of WPS on viral NA activity. NA activity from H1N1-GFP and H1N1-WT decreased significantly with WPS and zanamivir used as the positive control (**Figure 7A**). Moreover, WPS (1000 μg/mL) treatment had a significantly suppressive effect on NA activity for H3N2 (37.5%) and H1N1-WT (30%). The results suggest that WPS has additional inhibitory effects on the IAV release step through inhibiting NA, particularly at high concentrations.

### Inhibitory Effects of WPS on Virus Adsorption

IAVs can induce hemagglutination in RBCs. Therefore, we investigated whether WPS alters viral adsorption onto RBCs, resulting in hemagglutination. WPS decreased H3N2 viral titers by twofold in the HA assay (**Figure 7B**). However, WPS treatment did not inhibit RBC hemolysis in H1N1-infected cells (data not shown). These results suggest that the potent hemagglutination-inhibiting activity of WPS is attributed to a direct interaction with H3N2, particularly at high concentrations.

### HPLC Analysis of WPS

**FIGURE 8:**
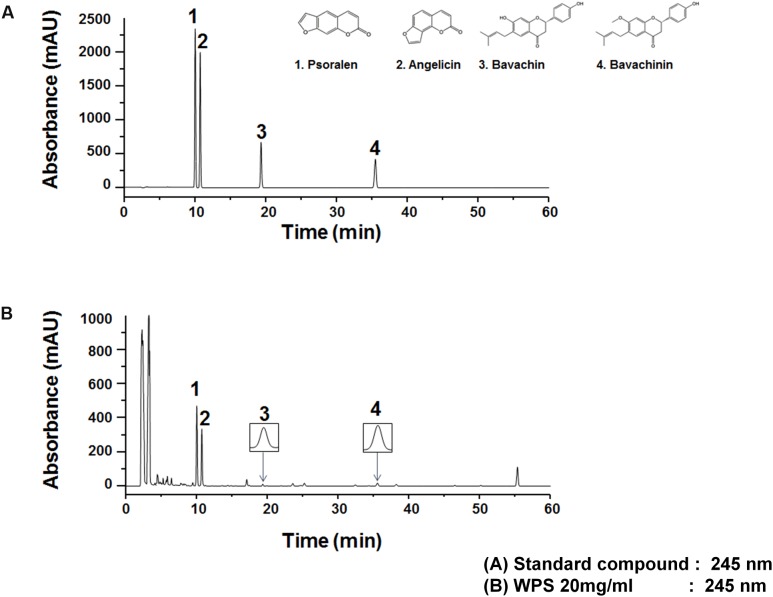
**Measurement of the representative component in WPS by high-performance liquid chromatography.** Psoralen (1), angelicin (2), bavachin (3), and bavachinin (4) were identified in the standard mixture **(A)** and WPS **(B)** at a wavelength of 245 nm. Data are representative of three independent experiments, and results were reproducible (**Supplementary Figure 1**).

The UV wavelength of chromatograms was adjusted on the basis of the maximum UV absorption wavelengths of the following major standard compounds: psoralen, 246 nm; angelicin, 248 nm; bavachin, 237 nm; and bavachinin, 237 nm; therefore, the analysis was performed at 245 nm. WPS constituents were determined by HPLC-diode array detection analysis, and each peak of the UV spectra was compared with that of representative standard compounds. As shown in **Figure 8**, HPLC-diode array detection analysis revealed a single peak specific for chemicals contained in WPS extracts at the following retention times: psoralen, 10.03 min; angelicin, 10.74 min; bavachin, 19.36 min; and bavachinin, 35.52 min. The following values were observed for the standard compounds: psoralen, 10.02 min; angelicin, 10.73 min; bavachin, 19.36 min; and bavachinin, 35.52 min (**Figure 8**).

## Discussion

Herbal medicines have gained popularity to control viral and bacterial infections; they have a low incidence of side effects (Martin and Ernst, 2003; Ehrhardt et al., 2007; Ge et al., 2010). Herbal extracts have anti-influenza effects (He et al., 2011; Haruyama and Nagata, 2013; Makau et al., 2013). In the present study, we demonstrated that WPS protects against infection by IAV subtypes in MDCK and RAW 264.7 cells. Psoralen and bakuchiol are the dominant compounds in WPS, and they have anti-viral activity against many viruses such as influenza and herpes simplex viruses (Nakashima et al., 1979; Redfield et al., 1981). Recently, bakuchiol was found to exert anti-influenza viral activity via Nrf2 activation (Shoji et al., 2015). However, no detailed mechanism has been described for the immunoregulatory and immune enhancing effects of WPS. Nevertheless, we were unable to define the mechanism of the anti-viral effects of WPS. WPS sufficiently protected against viral replication by modulating the innate immune response and inducing an anti-viral state. Moreover, we found that WPS enhanced the survival of IAV-infected MDCK and RAW 264.7 cells and inhibited influenza A viral infection and growth as well as viral protein expression (**Figures 2** and **6**). Innate immune cells, such as macrophages, initially recognize viral infections and rapidly induce the production of type I IFNs and pro-inflammatory cytokines, thereby generating anti-viral immune responses. We hypothesize that WPS induces an anti-viral state in murine macrophage cells by inducing anti-viral cytokines, modulating immune response, and inhibiting viral replication. Thus, we determined that type I IFN and IFN-stimulated gene levels (**Figures 3** and **4**) were altered in WPS-pre-treated RAW 264.7 cells. The effects of WPS on the phosphorylation of type I IFN signaling proteins, such as IRF3, TBK1, and STAT1, were examined in extract-treated RAW 264.7 cells. Type I IFNs upregulate the anti-viral status of infected cells (Sharma et al., 2003; Chiu et al., 2009). In our study, we found that WPS pre-treatment induced IRF3, TBK1, and STAT1 phosphorylation, thereby providing evidence of the downstream activation of signaling molecules in the type I IFN pathway.

This is the first study reporting that WPS possesses anti-influenza activity. We found that WPS chirality is important for this activity; thus, WPS should be considered a novel anti-H1N1 or -H3N2 drug. Our pre-incubation experiment illustrated that WPS more strongly inhibited H3N2 strains than H1N1 strains in HA and NA inhibition assays (**Figure 7**). This may reflect strain-associated differences in HA and NA viral proteins between the H3N2 and H1N1 strains, or the host cell response.

## Conclusion

We found that WPS can be a significant alternative anti-viral therapeutic agent for disrupting viral infection through the activation of type I IFN-mediated signaling, thereby inducing an anti-viral state in RAW 264.7 cells. Moreover, the anti-influenza effects of WPS are mediated by direct HA and NA inhibition. However, this study demonstrated that WPS had anti-viral activity only under *in vitro* conditions. Therefore, extensive future investigations for evaluating the potential of WPS for *in vivo* anti-viral therapeutic applications are warranted. An important step in this direction will be the identification of individual anti-viral agents from WPS and the analysis of their pharmacokinetic properties. In addition, specific signaling pathways and adequate dosage and efficacy duration of the extract in the host should be examined. The study results suggest that WPS has anti-viral activity against the influenza A subtypes H1N1 and H3N2. Our findings describe a possible mechanism of action of the anti-viral effects of WPS involving type I IFN signaling pathway activation and direct HA and NA inhibition. Thus, our results indicate that WPS has the potential to be an effective herbal remedy for the prophylaxis and treatment of influenza virus infection.

## Author Contributions

JYM and WKC developed the study design and revised the paper. JGC participated in the study design, performed the experiments, analyzed the data, and wrote the draft. YHJ, JHK, and TWO participated in the study design and analyzed the data. NHY conducted HPLC analysis. All authors have read and approved the final version of paper.

## Conflict of Interest Statement

The authors declare that the research was conducted in the absence of any commercial or financial relationships that could be construed as a potential conflict of interest.
