# Supplementary material for: In vitro Anti-viral Activity of Psoraleae Semen Water Extract against Influenza A Viruses
Source: Front Pharmacol. 2016 Nov 30;7:460. doi: 10.3389/fphar.2016.00460 (PMC5127801; doi:10.3389/fphar.2016.00460)
Supplement: Supplementary file 1 [file Data_Sheet_1.PDF]

**Supplementary Figure 1.**

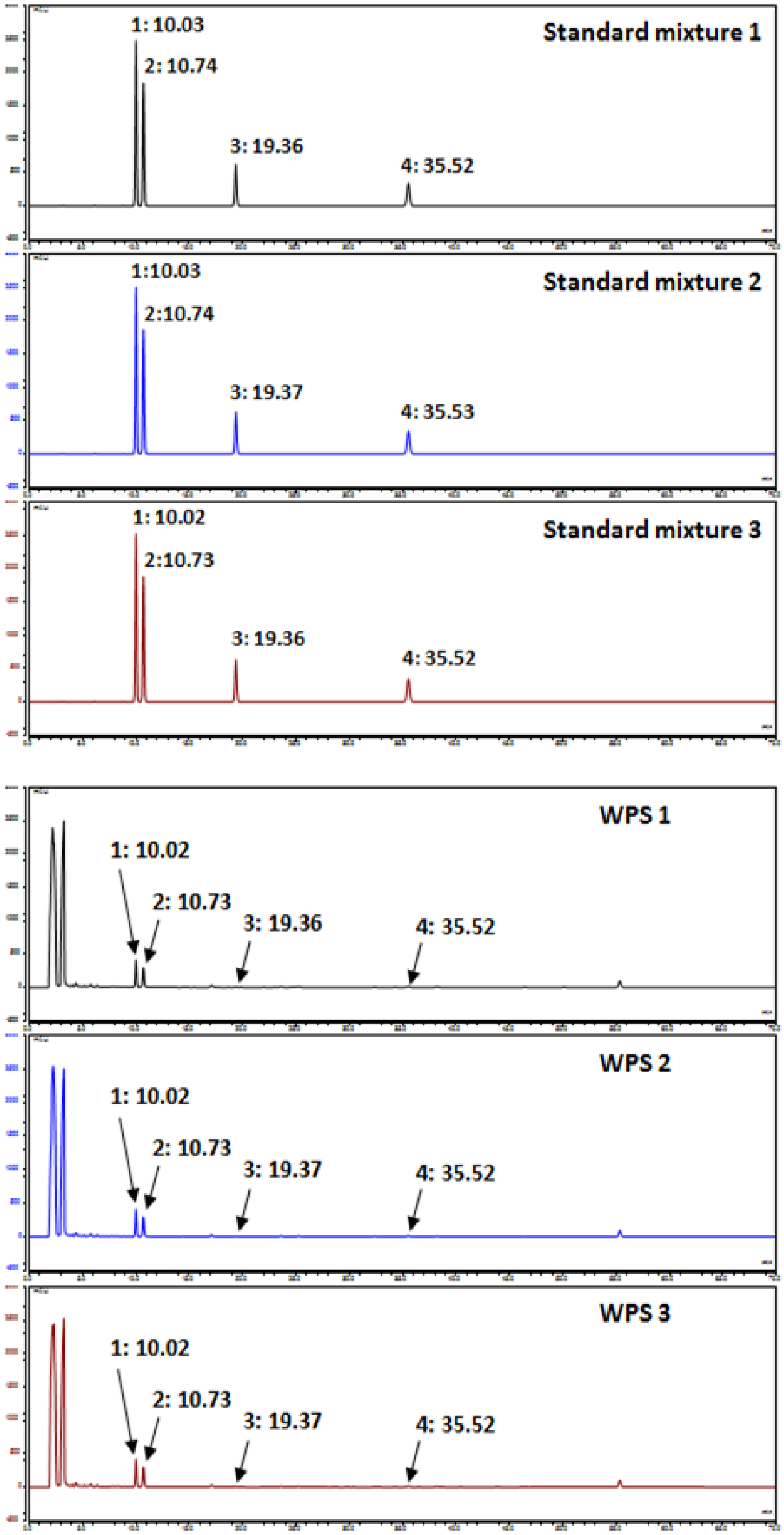


Supplementary Figure 1. Measurement of the representative component in the water extract of Psoraleae semen (WPS) by high-performance liquid chromatography. Psoralen (1), angelicin (2), bavachin (3), and bavachinin (4) were identified in the standard mixture (A) and WPS (B) at a wavelength of 245 nm. Data are representative of three independent experiments.
